# Supplementary material for: Forward and backward blocking in statistical learning
Source: PLoS One. 2024 Aug 5;19(8):e0306797. doi: 10.1371/journal.pone.0306797 (PMC11299817; doi:10.1371/journal.pone.0306797)
Supplement: S1 File — (DOCX) [file pone.0306797.s001.docx]

# Supplementary information 1

## Supplementary tables

## Table S1

*Posterior fixed effects of the post-hoc model of control condition on reaction times in Experiment 1. Estimate, estimation error, lower/upper limit of 95% profile credible intervals.*

| Predictors | *Estimate* | *Est. Error* | *CI (95%)* |
| --- | --- | --- | --- |
| Intercept | 491.09 | 9.73 | 472.16 – 510.23 |
| Expectation | 4.36 | 2.59 | -0.73 – 9.51 |
| Exposure | -30.03 | 4.33 | -38.34 – -21.45 |
| Expectation × Exposure | -2.02 | 3.71 | -9.26 – 5.21 |

## Table S2

*Posterior fixed effects of the post-hoc model of blocked condition on reaction times in Experiment 1. Estimate, estimation error, lower/upper limit of 95% profile credible intervals.*

| Predictors | *Estimate* | *Est. Error* | *CI (95%)* |
| --- | --- | --- | --- |
| Intercept | 485.56 | 9.70 | 466.60 – 504.68 |
| Expectation | 10.11 | 2.65 | 4.82 – 15.16 |
| Exposure | -27.38 | 3.94 | -35.05 – -19.58 |
| Expectation × Exposure | -0.95 | 3.68 | -8.26 – 6.25 |

## Table S3

*Posterior fixed effects of the post-hoc model of control condition on reaction times in Experiment 2. Estimate, estimation error, lower/upper limit of 95% profile credible intervals.*

| Predictors | *Estimate* | *Est. Error* | *CI (95%)* |
| --- | --- | --- | --- |
| Intercept | 500.48 | 9.47 | 481.97 – 519.28 |
| Expectation | 8.44 | 2.46 | 3.60 – 13.29 |
| Exposure | -22.24 | 4.70 | -31.37 – -12.93 |
| Expectation × Exposure | 0.38 | 3.57 | -6.43 – 7.36 |

## Table S4

*Posterior fixed effects of the post-hoc model of blocked condition on reaction times in Experiment 2. Estimate, estimation error, lower/upper limit of 95% profile credible intervals.*

| Predictors | *Estimate* | *Est. Error* | *CI (95%)* |
| --- | --- | --- | --- |
| Intercept | 499.01 | 9.42 | 480.60 – 517.78 |
| Expectation | 2.69 | 2.41 | -2.08 – 7.44 |
| Exposure | -22.72 | 4.38 | -31.25 – -13.85 |
| Expectation × Exposure | 3.40 | 3.79 | -4.11 – 10.83 |
