# Supplementary material for: Forward and backward blocking in statistical learning
Source: PLoS One. 2024 Aug 5;19(8):e0306797. doi: 10.1371/journal.pone.0306797 (PMC11299817; doi:10.1371/journal.pone.0306797)
Supplement: S2 File — (DOCX) [file pone.0306797.s002.docx]

# Supplementary information 2

# Supplementary Experiment 1

## Method

## Participants

The experiment was performed online using the Gorilla platform (Anwyl-Irvine et al., 2020), and participants were recruited through the Prolific platform (<https://www.prolific.co/>). 148 participants performed the experiment. 47 of them were excluded before they finished the experiment based on a priori exclusion criteria (see section ‘Exclusion and inclusion criteria’), and one participant was excluded from the final data analysis due to excessively slow responses (RTs above 3 times the mean absolute deviation [MAD] from the group mean). As a result, one hundred participants (37 females; mean age 24.49, range 18-40 years) were included in the data analysis. This final number of included participants was preregistered based on previous research (Richter & de Lange, 2019; Schmidt & De Houwer, 2019) considering that online data would be noisier and, therefore, a larger number of participants would be required to maintain the same statistical power. The pre-selected sample size yielded 84% power to detect a small sized (Cohen’s $d_{z}$ = 0.3) effect (α = 0.05).

All participants had normal or corrected to normal vision, normal hearing and no history of neurological or psychiatric conditions. They provided written informed consent and received financial reimbursement (8 euro per hour) for their participation in the experiment. The study followed the guidelines for ethical treatment of research participants by CMO 2014/288 region Arnhem-Nijmegen, The Netherlands.

## Experimental design

The design and procedure of Supplementary Experiment 1 was identical in all respects to Experiment 1 (see Figure S1a). In line with the traditional blocking paradigm Supplementary Experiment 1 comprised two training phases (training phase 1 and training phase 2) and a test phase. During the two training phases, leading object were perfectly predictive of their respective trailing object (i.e. P(trailing | leading = 1) ; see Figure S1b). Yet during the final test stage Expected and unexpected object pairs were presented equally often to prevent any learning (see Figure S1c). The main difference between Supplementary Experiment 1 and Experiment 1 is related to the type of leading stimuli and stimulus location. Leading stimulus was either a geometric shape or an everyday object. If the antedating leading stimulus was an object, then the blocked leading stimulus was a shape or vice versa. Both leading and trailing stimuli were presented at the center of the screen.

## Figure S1

*Experimental procedure and results of Experiment S1*

*Note.* (a) Experiment 1 comprised two training phases (training phase 1 and training phase 2) and a test phase. On every trial throughout the experiment, participants saw a pair of consecutively presented stimuli, i.e., a leading image followed by a trailing image. In training phase 1, the antedating leading stimulus (i.e., A), which could be either a shape or object, was followed by a specific trailing object. In training phase 2, a novel blocked leading stimulus (i.e., B) was presented in compound, along with the antedating (A) leading stimulus (i.e., AB), and followed by the same trailing object from the antedating stimulus in training phase 1. In addition, we introduced novel control compound leading (i.e., CD) and trailing (i.e., Y) stimuli. In the test phase, antedating, blocked or control leading stimuli were followed by the associated (expected) or not associated (unexpected) trailing object. Throughout the experiment, participants performed a categorization task on the trailing object. They reported, as fast as possible, whether the trailing object was electronic or non-electronic. (b) Statistical regularities depicted as image transition matrix with stimuli pairs in training phase 1 and training phase 2. Ls represent leading stimuli, and Ts represent trailing stimuli. (c) Statistical regularities depicted as image transition matrix with stimuli pairs in test phase. Green cells represent expected pairs, and red cells represent unexpected pairs. (d) Across participants' mean reaction times as a function of Expectation (expected / unexpected) and Condition (antedating / blocked / control). Participants responded faster to expected than unexpected trailing objects in each condition. There was no difference between blocked and control conditions. (e) Across participants' mean reaction time difference between expected and unexpected trials as a function of time. Please note that we split data into successive runs for visualization purposes only; data analysis was performed with number of trials as a continuous fixed factor (Exposure). Associations were rapidly extinguished during the test phase. Extinction was not different between conditions. (f) Posterior coefficient estimates of effects of the model jointly analyzing blocked and control conditions with error bars representing 95% confidence intervals. Estimates indicate significant results when they do not overlap with zero. (g) Across participants' proportion correct responses in pair recognition test. Participants showed slightly above chance-level performance in indicating whether the trailing object was likely or unlikely given the leading stimulus in all conditions.

## Data analysis

The data analysis of Supplementary Experiment 1 was identical in all respects to Experiment 1, except for the priors and the additional analysis of RT data *split by stimulus type in test phase*. The models were constructed using weakly informative priors. The prior distributions for the effects of interest were Gaussian distributions with zero mean and standard deviation adjusted to expected effect sizes: 50 for Expectation, 70 for Condition and 30 for Exposure, 70 for the interaction between Expectation and Condition. Further details and the complete model parametrization can be found in the R codes provided on the Donders Repository.

*Analysis of RT data split by stimulus type in test phase.* We conducted a follow-up analysis that tested for the effect of the type of leading stimulus (shape / object). We reasoned that leading object stimuli may have attracted more attention than leading shape stimuli, given that they were visually more salient than the surrounding grey shapes, and their category was task-relevant, as the task required object categorization on the trailing image. Given that associative learning depends on attention (Kruschke, 2001; Pacton & Perruchet, 2008), it was therefore conceivable that leading objects, rather than shapes, developed a stronger temporal association with trailing objects. We fit the model of antedating condition and the model of blocked and control conditions as described above, but with the inclusion of leading Stimulus Type (shape / object) as additional fixed factor. The model included a full random effect structure (i.e., a random intercept and slopes for all within-participant effects). If the posterior credible intervals of the interaction effects between Expectation and leading Stimulus Type did not overlap with zero, we run separate models for shapes and objects respectively, in order to test for the blocking effect for each stimulus type. The models were constructed using weakly informative priors centered at zero. All other analysis settings were as specified above.

## Results

*Analysis of RT data in test phase.* First, we compared the reaction times of expected and unexpected trials in the antedating condition to test whether repeated exposure to leading-trailing pairs led to learning their temporal association (see Table S1). We observed faster reaction times in expected (493 ms) than unexpected (508 ms) trials (b = 11.23, CI = [6.80, 15.59], Cohen’s $d_{z}$ = 0.54, see Figure S1d), indicating successful learning of stimulus transition probabilities and the consequent behavioral benefit of expectation in terms of response speed. In addition, we tested whether this behavioral benefit remained stable during the test phase or dwindled, as would be expected by extinction. In line with the latter, we observed an interaction effect between Expectation and Exposure (b = -9.28, CI = [-15.26, -3.38]), indicating that learning showed rapid extinction (expectation effect for run 1: 22 ms, run 2: 9 ms, run 3: 6 ms; see Figure S1e).

Next, we moved to our main question and tested for the presence of blocking (see Table S2 and Figure S1f). The reaction time difference between unexpected and expected trials was not different between control (11 ms) and blocked (12 ms) conditions (b = 1.85, CI = [-3.95, 7.51], Cohen’s $d_{z}$ = -0.04, see Figure S1d). This pattern of results presents suggest for the absence of blocking. There was also no difference in how the reaction time benefit for expected items behaved over time (b = -2.29, CI = [-11.17, 6.13]; expectation effect in blocked condition for run 1: 13 ms, run 2: 4 ms, run 3: 12 ms; expectation effect in control condition for run 1: 18 ms, run 2: 10 ms, run 3: 7 ms; see Figure S1e).

*Analyses of RT data split by stimulus type in test phase.* In a follow-up analysis, we tested whether the type of leading stimulus (shape / object) affected statistical learning. In the antedating condition (see Table S3), the reaction time difference between unexpected and expected trials was larger for leading object (20 ms) compared to leading shape (9 ms) trials according to the posterior CI (b = -10.00, CI = [-18.57, -1.48]), which indicated that object-object associations were somewhat stronger than shape-object associations. While the difference in RT was larger for object-object associations than shape-object associations, separate follow-up models showed that the reaction time difference was significant when the leading stimulus was an object (b = 15.19, CI = [7.98, 22.46], see Table S1 and Figure S2a-e), and it was still significant (b = 5.44, CI = [0.83, 10.05], Table S2 and see Figure S2b-f).

## Figure S2

*Results of Experiment S1 as a function of Stimulus Type*

*Note.* (a-b) Across participants' mean reaction times as a function of Expectation (expected / unexpected) and Condition (antedating / blocked / control) in leading objects (a) and leading shapes (b). The difference between expected and unexpected reaction times was larger for stimulus pairs with leading objects, compared to leading shapes. (c-d) Across participants' mean reaction time difference between expected and unexpected trials as a function of time in leading objects (c) and leading shapes (d). The decrease in reaction time difference between expected and unexpected trials over exposure showed rapid extinction in learning only in leading objects. (e-f) Posterior coefficient estimates of effects of the model jointly analyzing blocked and control conditions with error bars representing 95% confidence intervals in leading objects (e) and leading shapes (f). Estimates indicate significant results when they do not overlap with zero.

Across blocked and control conditions (see Table S4), the reaction time difference between unexpected and expected trials was also larger when the leading stimulus was an object (18 ms for B, 27 ms for D) compared to a shape (0 ms for B, 1 ms for D) (b = 18.40, CI = [11.52, 25.41]). Separate follow-up models showed that reaction times were faster in expected trials than in unexpected trials when the leading stimulus was an object (RT difference = 18 ms in blocked condition and 27 ms in control condition; b = 18.73, CI = [12.83, 24.5], see Table S3 and Figure S2a-e). This was not the case when the leading stimulus was a shape (RT difference = 0 ms in blocked condition and 1 ms in control condition; b = 0.11, CI = [-3.27, 3.44], Table S4 and see Figure S2b-f). Overall, the data suggest that shape – object associations could be learnt, but to a lesser extent than object – object associations. In particular, shape – object associations could be learnt only if a leading shape in isolation was followed by a trailing object (i.e., in the antedating condition), but not when the leading shape was concurrently paired with a leading object (in a compound stimulus) and then followed by the trailing object (i.e., in the blocked and control conditions). This pattern of results fits our prediction that leading objects attract more attention than shapes, given that they were visually more salient, and their category was task-relevant. As associative learning depends on attention (Kruschke, 2001; Pacton & Perruchet, 2008), this may have hampered associative learning between leading shapes and trailing objects. In other words, we found cue competition among the leading shape and object in the forms of overshadowing (Boddez et al., 2014; Pavlov, 1927; Schmidt & De Houwer, 2019), with the leading shape being overshadowed by the leading object. Finally, there was an interaction between Expectation, Condition and leading Stimulus Type (b = 4.09, CI = [-6.18, 15.80]), suggesting that the absence of blocking did not depend on leading Stimulus Type.

*Analyses of accuracy data in pair recognition test.* Participants showed slightly above chance-level performance in indicating whether the trailing object was likely or unlikely given the leading stimulus in the antedating (proportion correct = 58%; b = 0.32, CI = [0.23, 0.42])**,** blocked (proportion correct = 54%; b = 0.16, CI = [0.09, 0.24]) and control (proportion correct = 53%; b = 0.12, CI = [0.04, 0.19]) conditions (see Figure S1g). Response errors did not differ between the blocked and control conditions (b = -0.05, CI = [-0.15, 0.05]), indicating no blocking for the explicit knowledge of incidentally learned associations.

# Supplementary Experiment 2

Supplementary Experiment 1 showed that the type of leading stimulus critically influenced statistical learning. Antedating and control leading shapes got less strongly associated with the trailing object than antedating and control leading objects. Moreover, blocked and control leading shapes could not compete with the concurrent leading objects for associative strength because they attracted less attention. This imbalance between shapes and objects may provide an alternative explanation for the lack of blocking that we observed. Therefore, in Supplementary Experiment 2 we made one modification to our paradigm and only presented objects as leading and trailing stimuli to remove any potential difference in attention between different leading stimuli, which might finally result in a blocking effect.

## Method

## Participants

The experiment was performed online by using the Gorilla platform (Anwyl-Irvine et al., 2020), and participants were recruited through the Prolific platform (<https://www.prolific.co/>). 81 participants performed the experiment. 27 of them were excluded before they finished the experiment based on a priori exclusion criteria (see section ‘Exclusion and inclusion criteria’ above). Four extra participants were excluded from the final data analysis: two showed accuracy below 50% chance level in test phase; two showed overall excessively slow responses (RTs above 3 MAD from the group mean). As a result, fifty participants (16 females; mean age 23.90, range 18-34 years) were included in the data analysis, as preregistered. This final number of included participants was derived from the following a priori power calculation: we aimed for 90% power to detect the effect size of Cohen’s $d_{z}$ = 0.5 derived in the antedating leading object condition of Experiment 1 (α = 0.05).

All participants had normal or corrected to normal vision, normal hearing and no history of neurological or psychiatric conditions. They provided written informed consent and received financial reimbursement (8 euros per hour) for their participation in the experiment. The study followed the guidelines for ethical treatment of research participants by CMO 2014/288 region Arnhem-Nijmegen, The Netherlands.

## Experimental design

The design and procedure of Supplementary Experiment 2 was identical in all respects to Supplementary Experiment 1, apart from the type of leading stimuli and their location. Both leading and trailing stimuli were everyday objects. Leading and trailing objects were randomly presented on the left or right side of the central fixation point. Stimuli position (left / right) was counterbalanced with respect to Expectation (expected / unexpected) and Condition (antedating / blocked / control). In other words, leading and trailing objects appeared equally often on the left or right side of the central fixation point across trials. As a result, the expectation manipulation did not depend on spatial position. In addition, both hemi-fields were equally task-relevant, which fostered participants' attention to both sides.

**Data analysis**

The data analysis of Supplementary Experiment 2 was identical in all respects to Supplementary Experiment 1, except for omitting the factor Stimulus Type because this experiment featured only object stimuli (see Figure S3a).

**Figure S3**

*Experimental procedure and results of Experiment S2*

*Note.* (a) The design and procedure of experiment 2 was identical in all respects to experiment 1, apart from the fact that the leading stimulus was an object presented in the left or right side of the fixation point, and it was followed by the trailing object presented in the left or right side of the fixation point. (b) Across participants' mean reaction times as a function of Expectation (expected / unexpected) and Condition (antedating / blocked / control). Reaction times were faster to expected than unexpected trailing objects in blocked and control conditions. There was no difference between blocked and control condition in terms of reaction time difference between expected and unexpected trials, providing evidence for the absence of blocking effect. (c) Across participants' mean reaction time difference between expected and unexpected trials as a function of time. The decrease in reaction time difference between expected and unexpected trials over exposure showed rapid extinction in learning antedating condition. (d) Posterior coefficient estimates of effects of the model jointly analyzing blocked and control conditions with error bars representing 95% confidence intervals. Estimates indicate significant results when they do not overlap with zero. (e) Across participants' proportion correct responses in pair recognition test. Participants were not able to indicate above chance level whether the trailing object was likely or unlikely given the leading object in all conditions.

## Results

*Analyses of RT data in test phase.* First, we compared the reaction times of expected and unexpected trials in the antedating condition (see Table S5). We observed that reaction times for expected (503 ms) and unexpected (510 ms) trials, although showing a qualitative pattern similar to Experiment 1, were not significantly different from each other (b = 4.95, CI = [-0.07, 9.96], Cohen’s $d_{z}$ = 0.31, see Figure S3b). Therefore, unlike Experiment 1, our data do not provide robust support for learning of the conditional probabilities in condition A. There was however some statistical support for extinction, as the reaction time difference between expected and unexpected trials tended to decrease as the exposure increased (b = -8.17, CI = [-15.39, -0.91]) (expectation effect for run 1: 17 ms, run 2: 6 ms, run 3: 0 ms; see Figure S3c).

Next, we moved to our main question and compared reaction time differences between expected and unexpected stimulus pairs between B and C (see Table S6 and Figure S3d). The reaction time difference between unexpected and expected trials was not statistically different between control (8 ms) and blocked (1 ms) conditions (b = 3.34, CI = [-3.11, 9.85], Cohen’s $d_{z}$ = 0.24, see Figure S3b). Moreover, extinction was not different between B and C (b = 0.37, CI = [-9.60, 10.22]; expectation effect in blocked condition for run 1: 6 ms, run 2: -2, run 3: 0 ms; expectation effect in control condition for run 1: 11 ms, run 2: 4 ms, run 3: 5 ms; see Figure S3c).

*Analysis of accuracy data in pair recognition test.* Participants were not able to indicate above chance level whether the trailing object was likely or unlikely given the leading object in the antedating (proportion correct = 50%; b = 0, CI = [-0.15, 0.14]), blocked (proportion correct = 49%; b = -0.05, CI = [-0.17, 0.07]) or control (proportion correct = 50%; b = 0, CI = [-0.13, 0.14]) conditions (see Figure S3e).

## Supplementary tables

## Table S1

*Posterior fixed effects of the model of antedating condition on reaction times in Experiment 1. Estimate, estimation error, lower/upper limit of 95% profile credible intervals.*

| Predictors | *Estimate* | *Est. Error* | *CI (95%)* |
| --- | --- | --- | --- |
| Intercept | 502.44 | 8.42 | 485.21 – 518.66 |
| Expectation | 11.23 | 2.25 | 6.80 – 15.59 |
| Exposure | -15.14 | 3.51 | -22.08 – -8.19 |
| Expectation × Exposure | -9.28 | 3.01 | -15.26 – -3.38 |

## Table S2

*Posterior fixed effects of the model of blocked and control conditions on reaction times in Experiment 1. Estimate, estimation error, lower/upper limit of 95% profile credible intervals.*

| Predictors | *Estimate* | *Est. Error* | *CI (95%)* |
| --- | --- | --- | --- |
| Intercept | 494.45 | 8.34 | 478.02 – 510.93 |
| Expectation | 10.88 | 1.6 | 7.76 – 13.98 |
| Condition | 4.30 | 1.95 | 0.38 – 8.10 |
| Exposure | -19.10 | 3.08 | -25.19 – -13.08 |
| Expectation × Condition | 1.85 | 2.91 | -3.95 – 7.51 |
| Expectation × Exposure | -7.19 | 2.24 | -11.61 – -2.87 |
| Condition × Exposure | -3.00 | 2.26 | -7.49 – 1.40 |
| Expectation × Condition × Exposure | -2.29 | 4.48 | -11.17 – 6.13 |

## Table S3

*Posterior fixed effects of the model of antedating condition on reaction times split by stimulus type in Experiment 1. Estimate, estimation error, lower/upper limit of 95% profile credible intervals.*

| Predictors | *Estimate* | *Est. Error* | *CI (95%)* |
| --- | --- | --- | --- |
| Intercept | 502.27 | 8.42 | 485.68 – 518.72 |
| Expectation | 10.37 | 2.18 | 6.15 – 14.62 |
| Leading stimulus type | 56.95 | 5.6 | 46.13 – 67.99 |
| Exposure | -15.35 | 3.52 | -22.36 – -8.31 |
| Expectation × Leading stimulus type | -10.00 | 4.37 | -18.57 – -1.48 |
| Expectation × Exposure | -7.26 | 2.61 | -12.36 – -2.18 |
| Leading stimulus type × Exposure | 10.55 | 3.81 | 3.02 – 18.15 |
| Expectation × Leading stimulus type × Exposure | 1.12 | 5.38 | -9.32 – 11.81 |

## Table S4

*Posterior fixed effects of the model of blocked and control conditions on reaction times split by stimulus type in Experiment 1. Estimate, estimation error, lower/upper limit of 95% profile credible intervals.*

| Predictors | *Estimate* | *Est. Error* | *CI (95%)* |
| --- | --- | --- | --- |
| Intercept | 494.13 | 8.29 | 477.45 – 510.54 |
| Expectation | 9.30 | 1.65 | 6.05 – 12.49 |
| Condition | 4.58 | 1.96 | 0.71 – 8.47 |
| Leading stimulus type | -79.57 | 6.11 | -91.88 – -67.48 |
| Exposure | -19.54 | 3.03 | -25.49 – -13.66 |
| Expectation × Condition | 1.97 | 2.60 | -3.13 – 7.09 |
| Expectation × Leading stimulus type | 18.40 | 3.62 | 11.52 – 25.41 |
| Condition × Leading stimulus type | -6.78 | 3.88 | -14.36 – 0.89 |
| Expectation × Exposure | -5.79 | 1.90 | -9.53 – -2.06 |
| Condition × Exposure | -3.45 | 1.98 | -7.35 – 0.37 |
| Leading stimulus type × Exposure | -8.64 | 2.93 | -14.29 – -2.90 |
| Expectation × Condition × Leading stimulus type | 4.90 | 5.55 | -6.18 – 15.80 |
| Expectation × Condition × Exposure | -3.96 | 3.77 | -11.36 – 3.41 |
| Expectation × Leading stimulus type × Exposure | -17.54 | 3.70 | -24.82 – -10.32 |
| Condition × Leading stimulus type × Exposure | -0.21 | 3.74 | -7.41 – 7.13 |
| Expectation × Condition x Leading stimulus type × Exposure | 14.37 | 7.56 | -0.59 – 28.99 |

## Table S5

*Posterior fixed effects of the model of antedating condition on reaction times in Experiment 2. Estimate, estimation error, lower/upper limit of 95% profile credible intervals.*

| Predictors | *Estimate* | *Est. Error* | *CI (95%)* |
| --- | --- | --- | --- |
| Intercept | 512.83 | 18.97 | 475.52 – 549.47 |
| Expectation | 4.95 | 2.51 | -0.07 – 9.96 |
| Exposure | -18.40 | 4.29 | -26.74 – -10.02 |
| Expectation × Exposure | -8.17 | 3.70 | -15.39 – -0.91 |

## Table S6

*Posterior fixed effects of the model of blocked and control conditions on reaction times in Experiment 2. Estimate, estimation error, lower/upper limit of 95% profile credible intervals.*

| Predictors | *Estimate* | *Est. Error* | *CI (95%)* |
| --- | --- | --- | --- |
| Intercept | 515.30 | 19.42 | 476.89 – 533.55 |
| Expectation | 3.82 | 1.61 | 0.64 – 6.90 |
| Condition | -1.68 | 2.33 | -6.23 – 2.94 |
| Exposure | -21.29 | 4.38 | -29.92 – 12.70 |
| Expectation × Condition | 3.34 | 3.28 | -3.11 – 9.85 |
| Expectation × Exposure | -3.47 | 2.51 | -8.35 – 1.42 |
| Condition × Exposure | 1.12 | 2.58 | -3.86 – 6.15 |
| Expectation × Condition × Exposure | 0.37 | 5.08 | -9.60 – 10.22 |
