# Supplementary figures and images for: Forward and backward blocking in statistical learning
Source: PLoS One. 2024 Aug 5;19(8):e0306797. doi: 10.1371/journal.pone.0306797 (PMC11299817; doi:10.1371/journal.pone.0306797)

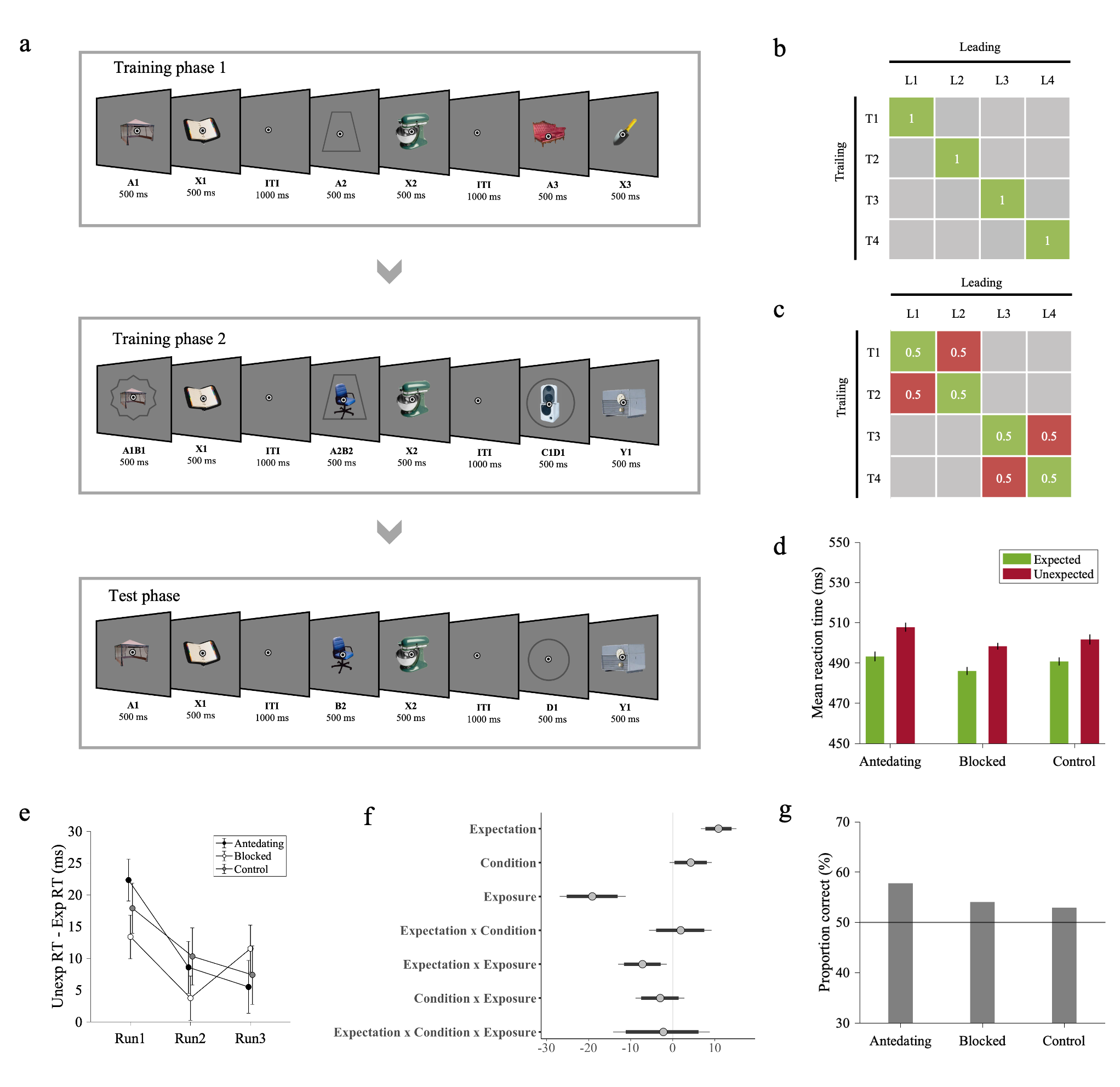

Supplement: S1 Fig — Experiment 1 comprised two training phases (training phase 1 and training phase 2) and a test phase. On every trial throughout the experiment, participants saw a pair of consecutively presented stimuli, i.e., a leading image followed by a trailing image. In training phase 1, the antedating leading stimulus (i.e., A), which could be either a shape or object, was followed by a specific trailing object. In training phase 2, a novel blocked leading stimulus (i.e., B) was presented in compound, along with the antedating (A) leading stimulus (i.e., AB), and followed by the same trailing object from the antedating stimulus in training phase 1. In addition, we introduced novel control compound leading (i.e., CD) and trailing (i.e., Y) stimuli. In the test phase, antedating, blocked or control leading stimuli were followed by the associated (expected) or not associated (unexpected) trailing object. Throughout the experiment, participants performed a categorization task on the trailing object. They reported, as fast as possible, whether the trailing object was electronic or non-electronic. (b) Statistical regularities depicted as image transition matrix with stimuli pairs in training phase 1 and training phase 2. Ls represent leading stimuli, and Ts represent trailing stimuli. (c) Statistical regularities depicted as image transition matrix with stimuli pairs in test phase. Green cells represent expected pairs, and red cells represent unexpected pairs. (d) Across participants’ mean reaction times as a function of Expectation (expected / unexpected) and Condition (antedating / blocked / control). Participants responded faster to expected than unexpected trailing objects in each condition. There was no difference between blocked and control conditions. (e) Across participants’ mean reaction time difference between expected and unexpected trials as a function of time. Please note that we split data into successive runs for visualization purposes only; data analysis was performed [file pone.0306797.s003.tif]

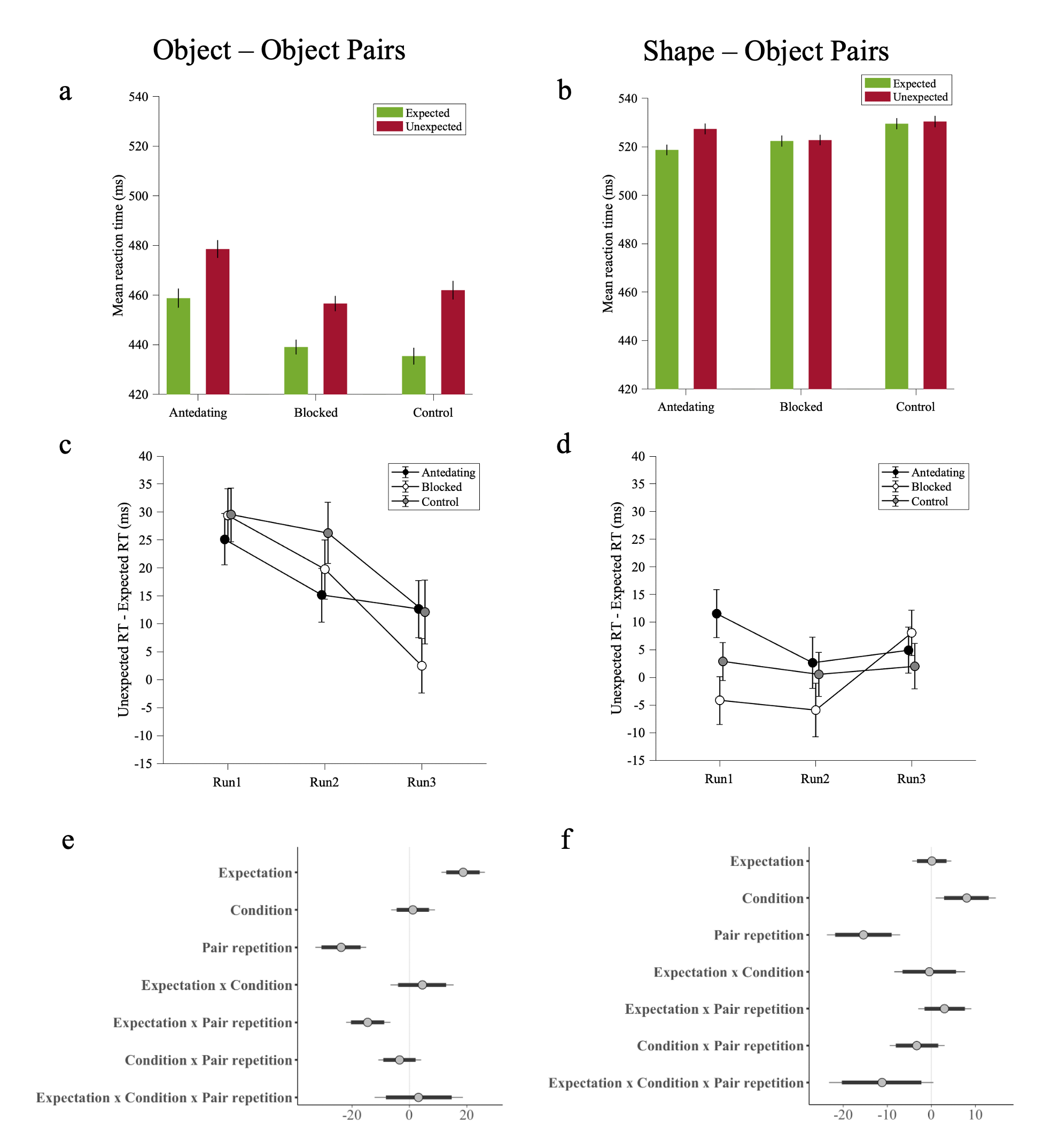

Supplement: S2 Fig — (a-b) Across participants’ mean reaction times as a function of Expectation (expected / unexpected) and Condition (antedating / blocked / control) in leading objects (a) and leading shapes (b). The difference between expected and unexpected reaction times was larger for stimulus pairs with leading objects, compared to leading shapes. (c-d) Across participants’ mean reaction time difference between expected and unexpected trials as a function of time in leading objects (c) and leading shapes (d). The decrease in reaction time difference between expected and unexpected trials over exposure showed rapid extinction in learning only in leading objects. (e-f) Posterior coefficient estimates of effects of the model jointly analyzing blocked and control conditions with error bars representing 95% confidence intervals in leading objects (e) and leading shapes (f). Estimates indicate significant results when they do not overlap with zero. (TIF) [file pone.0306797.s004.tif]

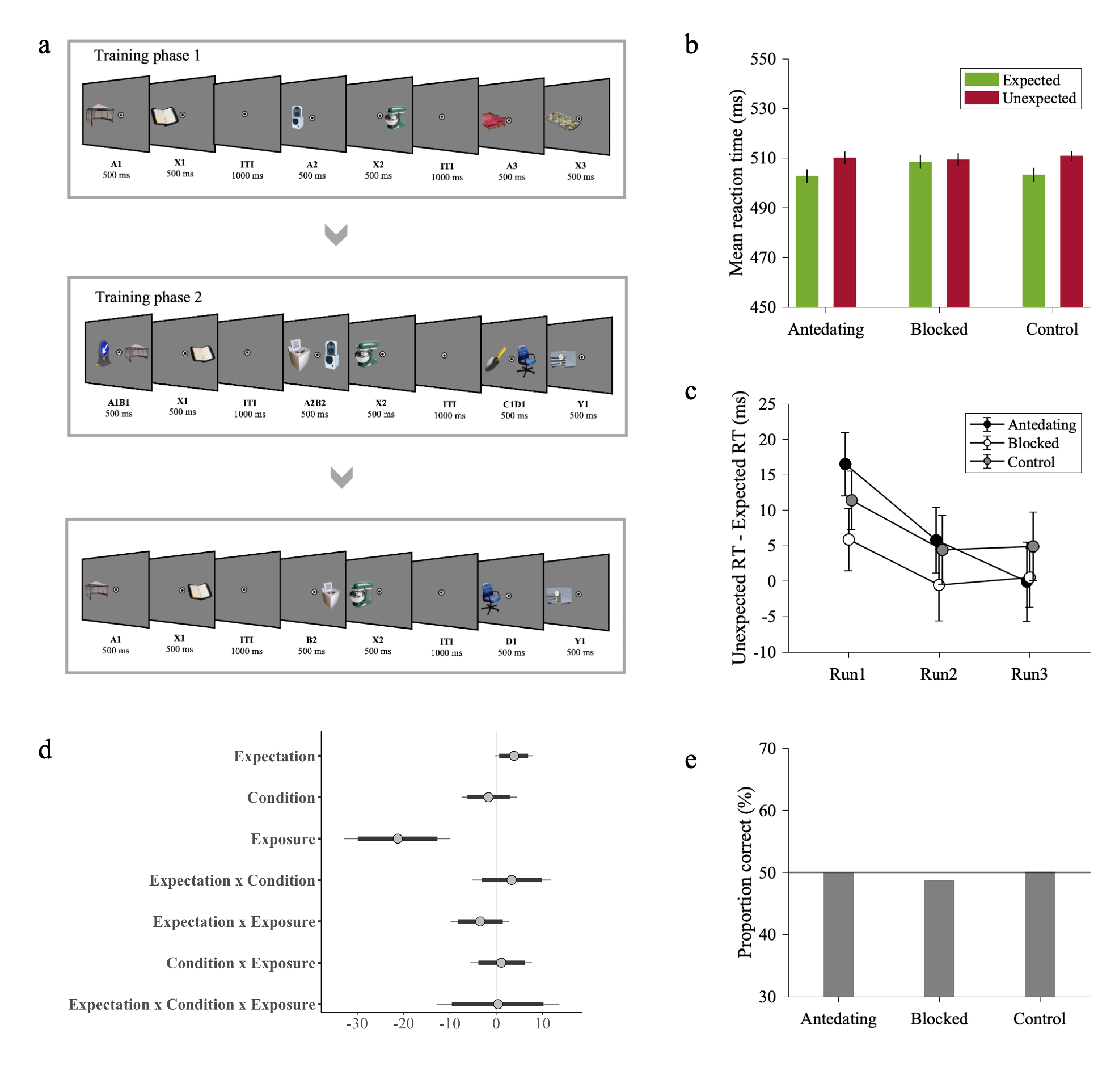

Supplement: S3 Fig — (a) The design and procedure of experiment 2 was identical in all respects to experiment 1, apart from the fact that the leading stimulus was an object presented in the left or right side of the fixation point, and it was followed by the trailing object presented in the left or right side of the fixation point. (b) Across participants’ mean reaction times as a function of Expectation (expected / unexpected) and Condition (antedating / blocked / control). Reaction times were faster to expected than unexpected trailing objects in blocked and control conditions. There was no difference between blocked and control condition in terms of reaction time difference between expected and unexpected trials, providing evidence for the absence of blocking effect. (c) Across participants’ mean reaction time difference between expected and unexpected trials as a function of time. The decrease in reaction time difference between expected and unexpected trials over exposure showed rapid extinction in learning antedating condition. (d) Posterior coefficient estimates of effects of the model jointly analyzing blocked and control conditions with error bars representing 95% confidence intervals. Estimates indicate significant results when they do not overlap with zero. (e) Across participants’ proportion correct responses in pair recognition test. Participants were not able to indicate above chance level whether the trailing object was likely or unlikely given the leading object in all conditions. (TIF) [file pone.0306797.s005.tif]
